# Identification of Nuclear Localization Sequence (NLS) Sites in R2R3-MYB Transcription Factor Involved in Anther Development

**DOI:** 10.3390/cells14070470

**Published:** 2025-03-21

**Authors:** Si-Da Zhou, Que Zhou, Yan-Dan Cui, Xiang Zhong, Xing Chen, Xue-Rong Lin, Zhong-Nan Yang, Jun Zhu

**Affiliations:** 1 Shanghai Key Laboratory of Plant Molecular Sciences, College of Life Sciences, Shanghai Normal University, Shanghai 200234, China; sida.zhou@uni-potsdam.de (S.-D.Z.); zhouque@shnu.edu.cn (Q.Z.); 1000526268@smail.shnu.edu.cn (Y.-D.C.); 1000497593@smail.shnu.edu.cn (X.Z.);; 2 Development Center of Plant Germplasm Resources, College of Life Sciences, Shanghai Normal University, Shanghai 200234, China; 3 Institute for Biochemistry and Biology, University of Potsdam, Karl-Liebknecht-Str. 24-25, 14476 Potsdam-Golm, Germany

**Keywords:** nuclear localization sequence, R2R3-MYB transcription factor, anther development

## Abstract

The R2R3-MYB family of transcription factors (TFs) plays a crucial role in cell specification and secondary metabolism regulation during plant development. In Arabidopsis, MS188, a typical R2R3-MYB protein, is essential for tapetal development and pollen wall formation. However, the nuclear localization sequence (NLS) responsible for directing MS188 into the nucleus has not been fully elucidated. In this study, the subcellular localization of the NLS-containing proteins was determined by GFP tagging in tobacco leaves, and three NLS regions within MS188 were identified: two located at the N-terminus of R2-MYB and one at the C-terminus of R3-MYB. We further narrowed the NLSs located at amino acids (AAs) 12–15, 18–22, and 96–107 via point mutation analysis. Combined with the cytoplasmic protein FBA6, these NLSs fusion proteins could localize in the nucleus. Importantly, the proteins with mutations in AAs 18–22 exhibited completely cytoplasmic signals, whereas other mutated sites partially abolished the nuclear signals. These findings suggest that the NLS at AAs 18–22 is sufficient for nuclear localization. To confirm the NLS functions in vivo, we constructed the vectors including the *MS188* gene without the NLS sites, which failed to complement the male sterile phenotype of *ms188*. We also searched the highly conserved NLSs in other R2R3-MYB TFs and showed they are required for nuclear localization. Collectively, these findings revealed the specific NLS regions within R2R3-MYB transcription factors and highlighted their critical role for subcellular localization in plant developmental regulation.

## 1. Introduction

Transcription factors (TFs) are trans-acting DNA-binding proteins that bind to specific DNA sequences and control genetic information from DNA to mRNA via the regulation of transcription. TFs contain four regions: DNA-binding domains (DBDs), transcription regulation domains (including the activation domain and/or suppressor domain), oligomerization sites, and nuclear location sequences (NLSs) [1,2]. MYB (myeloblastosis) proteins are transcription factors that were originally identified in the avian myeloblastosis virus and can be found in all eukaryotes [3]. The MYB domain generally consists of two or three imperfect amino acid sequence repeats, and each repeat contains 50–53 amino acids, forming a helix-turn-helix (HTH) motif [4]. This HTH structure provides a hydrophobic core for DNA binding [5]. The c-Myb protein, the oncogene homologue of the v-myb myeloblastosis viral protein, has three repeats, named R1, R2, and R3, and the repeats of other MYB proteins have since been identified on the basis of their sequence similarities [6]. In plants, R2R3-MYB TFs belong to the largest subfamily of the MYB superfamily and are expressed in most plants. Most genes encoding R2R3-MYB genes are thought to have evolved from an R1R2R3-MYB gene ancestor; the R1 repeat was lost, and the gene family subsequently expanded [4].

R2R3-MYBs have been studied and shown to be widely involved in plant organ and tissue development, metabolism, and responses to stresses through genetic approaches [7]. Among them, many R2R3-MYB proteins are well known to function in the regulation of secondary metabolism, including the benzenoid, phenylpropanoid, terpenoid, and glucosinolate pathways [8,9,10,11]. In particular, R2R3-MYBs associated with the biosynthesis pathways of phenylpropanoid-derived compounds (monolignols, flavonoids, phenolic acids, and stilbenes) have been well studied [8]. For example, the PAP1/PAP2/WD-repeat (WDR)/basic-helix-loop-helix (bHLH) complex regulates anthocyanin biosynthesis in Arabidopsis [12,13]. In the flavonol biosynthesis pathway, genes encoding chalcone synthase (CHS), chalcone isomerase (CHI), flavanone 3-hydroxylase (F3H), and flavonol synthase (FLS) are activated by AtMYB11, AtMYB12, and AtMYB111 [14,15]. In these biosynthesis pathways, some R2R3-MYB proteins are activators, whereas others are repressors [16]. In addition, AtMYB30, AtMYB60, and AtMYB15 contribute to the drought stress response and disease resistance [17,18,19]. Several R2R3-MYB proteins have been characterized to function in developmental processes. For example, AtMYB23 participates in a positive feedback loop to regulate trichome cell differentiation and precisely establish the root epidermal pattern [20,21]. AtMYB37, AtMYB28, and AtMYB84 play redundant roles in axillary meristem formation [22,23]. AtMYB91/AS1 competes with KNOX proteins to regulate shoot morphogenesis and leaf patterning, determining leaf dorsiventral polarity [24].

R2R3-MYB proteins also play essential roles in pollen development. In Arabidopsis anthers, several tapetum-expressed TFs constitute a regulatory pathway (DYT1-TDF1-AMS-MS188-MS1) [25]. In this pathway, TDF1 and MS188/MYB80 belong to the R2R3-MYB family. TDF1 regulates tapetal cell differentiation and function [26]. TDF1 was later found to interact with ABORTED MICROSPORE (AMS), a bHLH TF, and form a TDF1–AMS complex to regulate tapetum development via a feed-forward loop [27]. MS188 is a key TF required for tapetal development and pollen outer wall (exine) formation [28]. Similarly, the AMS-MS188 complex controls the sporopollenin biosynthesis process through a feed-forward loop for exine formation [29,30]. Previous studies have shown that TDF1 and MS188 bind to specific promoter regions of downstream genes and activate the transcription of target genes [27,30,31], indicating that both TFs are required for transcription in the nucleus.

The entry of transcription factors into the nucleus is essential for their function, and the active transport of proteins in the nucleus is determined mainly by the interaction between nuclear localization signals (NLSs) in cargo proteins and the importin α/ß complex [32,33]. Importin α/ß recognizes two types of NLSs, known as classical NLSs (cNLSs). The NLS is a short amino acid sequence that is divided into monopartite cluster types and bipartite cluster types. The typical nuclear localization sequence of nuclear localization proteins consists of a short amino acid sequence that contains one or two clusters of basic amino acids (126P-K-K-K-R-V132). This sequence was first discovered in the simian virus 40 (SV40) large T antigen, which allows the protein to localize to the nucleus [34,35]. It is considered the prototypical monotypic NLS and is found in plants, indicating the conservation of the nuclear transport mechanism between animals and plants. Bipartite cluster-type NLSs have a second nuclear localization sequence of 10–12 amino acid residues downstream of the first sequence.

TFs, TDF1, and MS188 are presumed to enter the nucleus and execute their functions; however, their NLSs are still not fully understood. Here, we defined the NLS of MS188, a bipartite cluster-type NLS located at the two ends of the R2R3-MYB domain. The findings of this study suggest that the R2R3-MYB domain facilitates not only DNA binding but also the nuclear localization of these TFs. We further identified the precise NLS at amino acids (AAs) 12–15, 18–22, and 96–107 of MS188, with high similarity to TDF1 and MYB2 NLSs. The genetic evidence revealed that MS188 without these NLS sites failed to complement the male sterile phenotype of an MS188 mutant. Therefore, these NLSs can guide nuclear translocation and have a conserved function in the R2R3-MYB family.

## 2. Materials and Methods

### 2.1. Plant Materials and Growth Conditions

All Arabidopsis (*Arabidopsis thaliana*) plants, including the wild-type, *ms188*, and transgenic lines, were in the *Columbia-0* background. The seeds were sown on vermiculite and allowed to imbibe for 3 d at 4 °C. These plants were cultivated under long-day conditions (16 h of light and 8 h of dark) in a plant cultivation room maintained at approximately 22 °C. The transgenic plants were produced through *Agrobacterium tumefaciens*-mediated transformation. Tobacco plants (*Nicotiana benthamiana*) were grown in the same cultivation room under the same photoperiod and temperature conditions for approximately 4 weeks before being used.

### 2.2. Plasmid Construction and Transient Expression Analysis

*MS188* truncations were fused to *p35S-GFP* using the pEASY Uni-Seamless Cloning and Assembly Kit according to the manufacturer’s instructions and transformed into *Agrobacterium strain* GV3101 (the sequences of the primers used are listed in Table S1). *Agrobacterium* was cultured to an OD_600_ of approximately 1.2–1.5. The bacterial cells were then pelleted and resuspended in MES/MgCl_2_/AS solution (10 mM MES, pH 5.6, 10 mM MgCl_2_, 0.5 mM acetosyringone) to a final OD_600_ of 1.0 and infiltrated into young tobacco leaves. After approximately 36–48 h, the subcellular localization of the MS188 protein was determined using an Olympus FV3000 laser scanning microscope (Olympus, Tokyo, Japan).

The *MS188*, *TDF1,* and *MYB2* genes were mutated via homologous overlap and further fused to *p35S-GFP* using the pEASY Uni-Seamless Cloning and Assembly Kit. The constructs were transformed into *Agrobacterium* strain GV3101, and the bacterial cells were cultured overnight and resuspended in MES/MgCl_2_/AS solution for transformation. Subcellular localization of the mutated MS188, TDF1m and MYB2 was observed under the same microscope.

### 2.3. Genetic Complementation Experiment

The *MS188* CDS fragments with the corresponding mutations, driven by the *MS188* native promoter (1554 bp), were amplified using the gene-specific primers (the sequences of primers used are listed in Table S1). The resulting PCR products were cloned and inserted into the pCAMBIA1300 binary vector (CAMBIA, Canberra, Australia), transformed into *Agrobacterium strain* GV3101, and subsequently introduced into *ms188*^+/−^ heterozygous lines using the floral-dip method. Seeds were selected on 1/2 MS media plates supplemented with 20 mg/L hygromycin B (Roche, Basel, Switzerland). The T1 lines were genotyped to identify *ms188*^−/−^ homozygous plants.

### 2.4. Cytological Analysis

Flower buds at various developmental stages were subjected to fixation in FAA solution overnight. The FAA solution consisted of 50% (*v*/*v*) ethanol, 5.0% (*v*/*v*) acetic acid, and 3.7% (*v*/*v*) formaldehyde. The fixed flower buds were then dehydrated through a series of graded ethanol solutions. After dehydration, the flower buds were transferred to xylene and then embedded in Spurr’s epoxy resin (Ted Pella, Redding, CA, USA). Using glass knives, thin sections of 1 μm thickness were cut on a PowerTome XL (manufactured by RMC Products, Tucson, AZ, USA). Once stained with toluidine blue, the sections were rinsed three times with pure water and allowed to air dry. Finally, bright-field photographs of the anther cross-sections were captured using an Olympus DX51 digital camera (Olympus, Tokyo, Japan).

### 2.5. Phylogenetic Analysis

The full-length protein sequences of MS188, TDF1, and MYB2 were subjected to multiple sequence alignments using ClustalW. The presentation of the alignment results was prepared using Boxshade (https://www.genome.jp/tools-bin/clustalw, accessed on 6 January 2024).

### 2.6. Confocal Microscopy

All fluorescence images were obtained using an Olympus FV3000 laser scanning microscope (Olympus, Tokyo, Japan). For monitoring the GFP signal, a 488 nm laser line was used for excitation, and a 498–532 nm bandpass filter was used for detection [36].

## 3. Results

### 3.1. The NLS Is Localized in the N-Terminus of MS188

**Figure 1:**
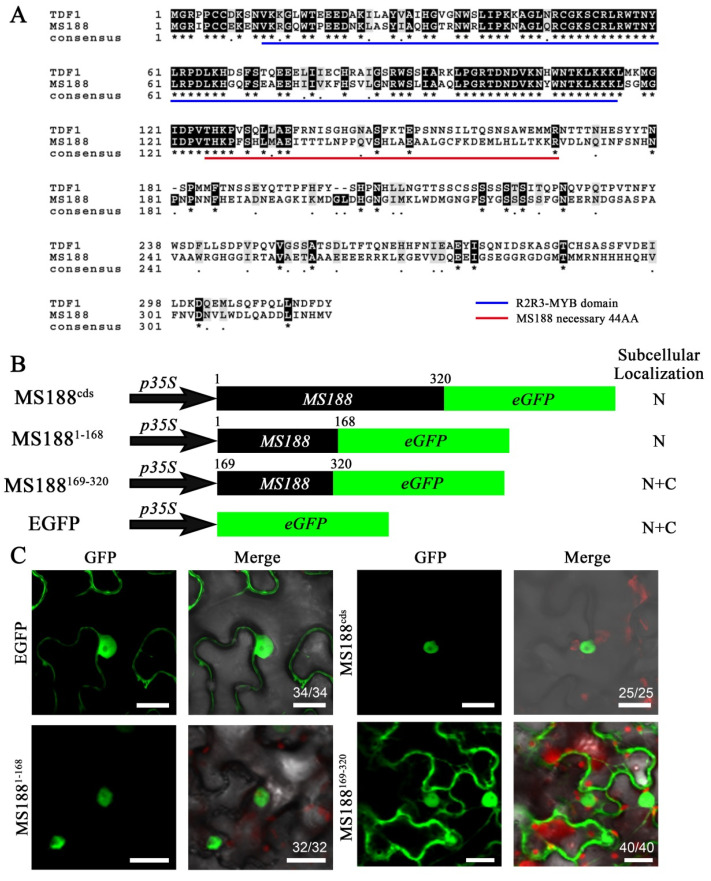
MS188^1–168^-GFP is located in the nucleus. (**A**) Amino acid sequence alignment of two R2R3-MYB genes: MS188 and TDF1. The R2R3 domain is marked by the blue bar, and the necessary 44 AAs of MS188 are marked by the red bar. Dark shading, identical residues; light shading, similar residues. * represents the identical animo acids between two sequences. (**B**,**C**) The complete MS188^cds^ and two fragments, AAs 1–168 and AAs 169–320, were fused with GFP and infiltrated into tobacco leaves. MS188^cds^ and MS188^1–168^-GFP were detected only in the nucleus. MS188^169–320^-GFP was distributed throughout the cytoplasm and the nucleus. N, nucleus; C, cytoplasm. Bars = 20 μm. The number in the bottom right corner of the Merge panel represents the statistical number of observations.

Although MS188 has been shown to be localized in the nucleus [28,37,38], its precise NLS motif in this protein remains unknown. In this study, protein sequence alignment between MS188 and TDF1 revealed that the N-terminal region, including the R2R3-MYB domain, was conserved rather than the C-terminal region (Figure 1A). Adjacent to the R2R3-MYB domain, there is a 44 amino acid sequence that appears to be necessary for MS188/MYB80 function [37]. The activation domain is located in the last 62 amino acid residues of the C-terminal region [39]. On the basis of this information, we further divided the MS188 protein into two parts, spanning amino acid residues 1–168 (MS188^1–168^) and 169–320 (MS188^169–320^). We constructed a GFP construct on full-length MS188, MS188^1–168^, and MS188^169–320^, driven by the 35S promoter (Figure 1B). A transient expression assay was performed in which the constructs were transferred into tobacco leaves (*N. benthamiana*). The subcellular locations of the GFP signals were examined in the transfected cells at 48 h post-transfection. The results revealed that the full-length MS188-GFP protein was located in the nucleus of tobacco leaf cells, which was consistent with the findings of a previous study [28] (Figure 1C). For the constructs, *MS188^169–320^-GFP* was distributed in both the nucleus and cytoplasm, which is similar to the expression pattern of 35S-GFP (Figure 1C). However, the *MS188^1–168^-GFP* signal was solely detected in the nucleus (Figure 1C). This result indicated that the NLS region of MS188 is located within amino acid residues 1–168 in the N-terminal region.

### 3.2. MS188 Contains a Bipartite Cluster-Type NLS

**Figure 2:**
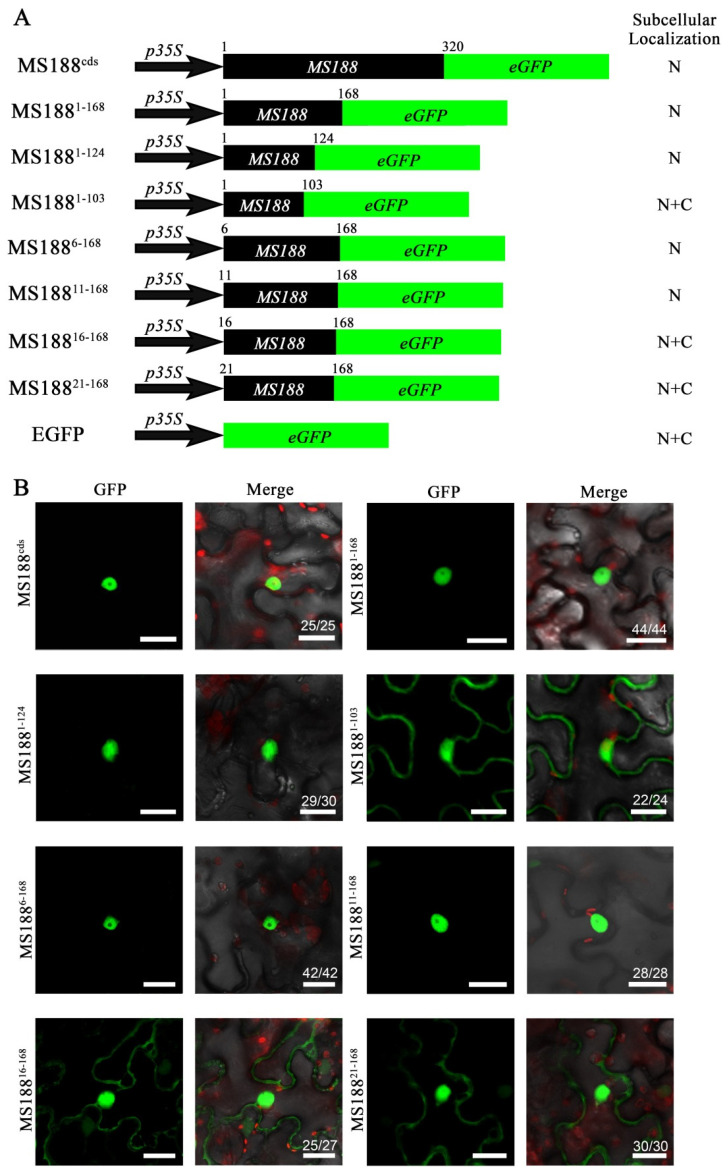
MS188 has NLSs at both the N- and C-termini of the R2R3-MYB domain. (**A**) To determine the NLS of MS188, MS188^1–168^ was truncated into additional short segments: MS188^1–168^, MS188^1–124^, MS188^1–103^, MS188^6–168^, MS188^11–168^, MS188^16–168^, and MS188^21–168^ fusions with GFP were infiltrated into tobacco leaves. N, nucleus; C, cytoplasm. (**B**) The fluorescence of MS188^1–168^-GFP, MS188^1–124^-GFP, MS188^6–168^-GFP, and MS188^11–168^-GFP was detected only in the nucleus. MS188^1–103^-GFP, MS188^16–168^-GFP, and MS188^21–168^-GFP were distributed throughout the cytoplasm and the nucleus. Bars = 20 μm. The number in the Merge panel represents the statistical number of observations.

To identify the NLS sites of MS188 in detail, we further constructed shorter truncations of MS188, specifically from the MS188^1–168^ region, fused with GFP. We constructed MS188^1–168^-GFP, MS188^1–124^-GFP, and MS188^1–103^-GFP fusions (C-terminal truncations) and transfected them into tobacco leaves (Figure 2A). After transfection, MS188^1–168^ and MS188^1–124^ were detected to be localized in the nucleus (Figure 2B), indicating that the NLS site was in the region of residues 1–124. However, the MS188^1–103^-GFP signal was observed in both the nucleus and the cytoplasm (Figure 2B). The results suggested that amino acid residues 104–124 of MS188 contain the putative NLS. We also prepared the N-terminal truncations MS188^6–168^-GFP, MS188^11–168^-GFP, MS188^16–168^-GFP, and MS188^21–168^-GFP (Figure 2A). MS188^6–168^-GFP and MS188^11–168^-GFP showed nuclear localization, while MS188^16–168^ and MS188^21–168^ signals were detected both in the nucleus and cytoplasm (Figure 2B), indicating that amino acids 11–15 also contain a putative NLS. These results suggested that MS188 has a bipartite cluster-type NLS with two motifs in AAs 11–15 and 104–124.

### 3.3. The NLSs of MS188 Include Amino Acid Residues 12–15, 18–22 and 96–107

**Figure 3:**
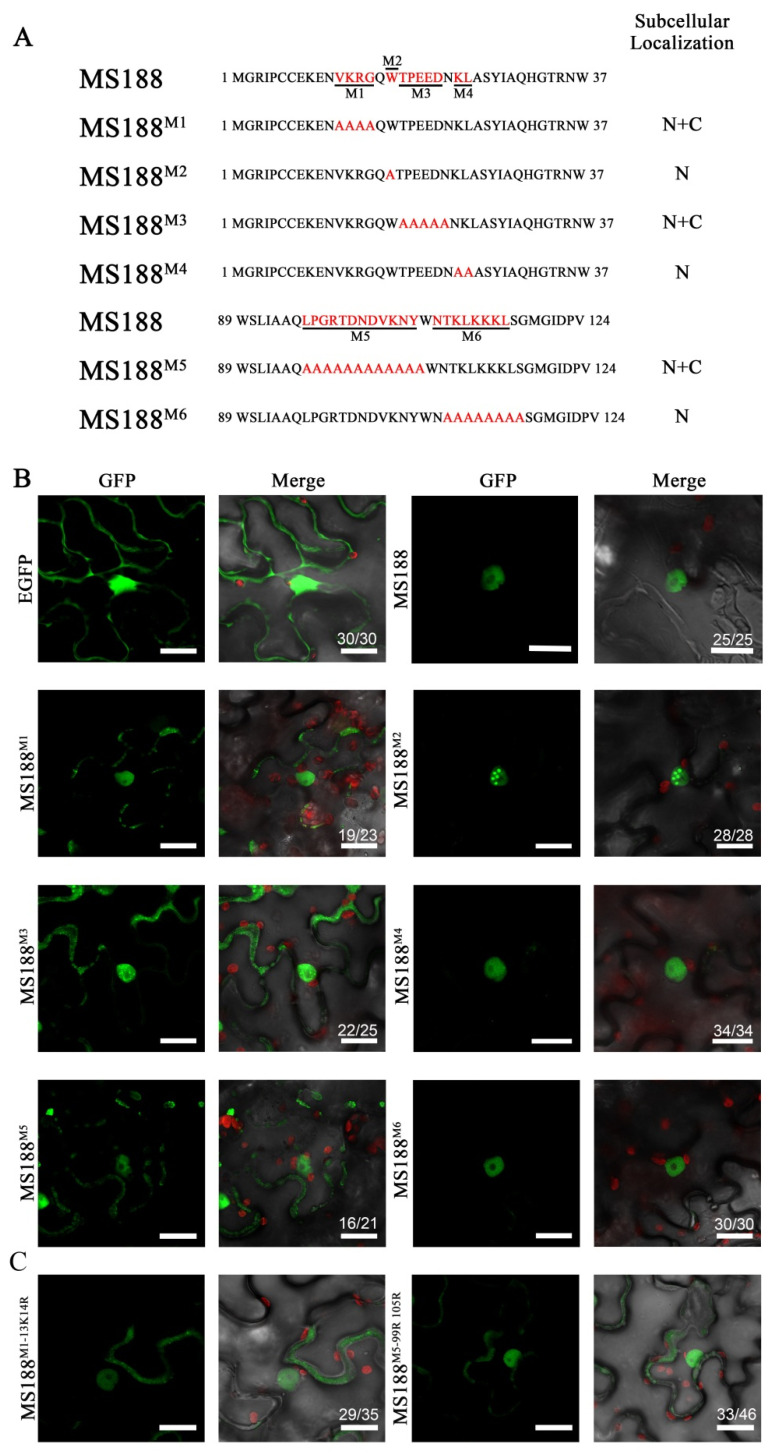
MS188^12–15^, MS188^18–22^, and MS188^96–107^ contribute to MS188 nuclear localization. (**A**) Point mutation of MS188 was performed to refine the NLS of MS188. The selected amino acids are shown in red font and were mutated to alanine (**A**). M1: 12VKRG15; M2: W17; M3: 18TEEED22; M4: 24KI25; M5: 96LPGRTDNDVKNH107; M6: 109NTKLKKKL116. All the mutated proteins were fused with GFP and infiltrated into tobacco leaves. N, nucleus; C, cytoplasm. (**B**) The fluorescence of MS188^M2^-GFP, MS188^M4^-GFP, and MS188^M6^-GFP was detected only in the nucleus. MS188^M1^-GFP, MS188^M3^-GFP, and MS188^M5^-GFP were distributed throughout the cytoplasm and the nucleus. Bars = 20 μm. (**C**) The fluorescence of MS188^M1–13K14R^-GFP and MS188^M5–99R105R^-GFP was distributed throughout the cytoplasm and the nucleus. Bars = 20 μm. The number in the Merge panel represents the statistical number of observations.

To accurately identify the specific NLSs, we used the point mutation approach to mutate the candidate bases into alanine (A). On the basis of the above results, we mutated the highly conserved amino acid sequences near the above two motifs. A total of six amino acid sequences were constructed for tobacco transient transformation (Figure 3A). In MS188^M1^, 12VKRG15, a region highly conserved in the R2R3 domain, is mutated to 12AAAA15. Transient expression assays revealed that the GFP signal of MS188^M1^ appeared in both the nucleus and the cytoplasm, indicating that these four amino acids are part of the NLS (Figure 3B). MS188^M2^ (W17A) is a mutant of the tryptophan at position 17, which is involved in the formation of the first α helix of the MYB domain. The MS188^M2^ GFP signal was still nuclear localized, indicating that the nuclear localization of MS188 may not depend on its first α helix (Figure 3B). In MS188^M3^, 18TEEED22, a highly conserved portion of the R2R3 domain, is mutated to 18AAAAA22. The GFP signal of MS188^M3^ was observed in the nucleus and cytoplasm, indicating that these five amino acids also contained NLS components (Figure 3B). There are at least three acidic amino acids at these sites, indicating that they also play a major role in the process of nuclear entry. In MS188^M4^, 24KI25 is mutated to 24AA25. The results revealed that the nuclear localization signal did not change, indicating that these sites are not involved in the composition of the NLS (Figure 3B). The MS188^M5^ mutant contains the sequence 96LPGRTDNDVKNH107, which is highly conserved in Arabidopsis R2R3 family members. The fluorescence of MS188^M5^-GFP was present in the nucleus and cytoplasm, indicating that these sites participated in the formation of the NLS (Figure 3B). MS188^M6^ contains the region 109NTKLKKKL116, which contains many basic amino acid residues. However, the MS188^M6^-GFP signal showed nuclear localization, indicating that these sites did not participate in the composition of the NLS (Figure 3B). Taken together, these results allow us to conclude that amino acid residues 12–15, 18–22, and 96–107 are involved in NLS composition and that acidic amino acids are also required for the process of nuclear entry. Previous studies have indicated that basic amino acids are important for nuclear localization sequences [40,41]. To verify the significance of these basic amino acids, we further mutated the basic amino acid sites 13 K and 14R in the MS188^M1^ region and the two basic amino acid sites 100 K and 106 K in the MS188^M5^ region. As a result, the protein diffused into the cytoplasm (Figure 3C). Therefore, basic amino acids in these regions of MS188 are required for nuclear localization.

### 3.4. Basic Amino Acid Residues Play Important Roles in the NLS Sites of MS188 for Nuclear Transportation

**Figure 4:**
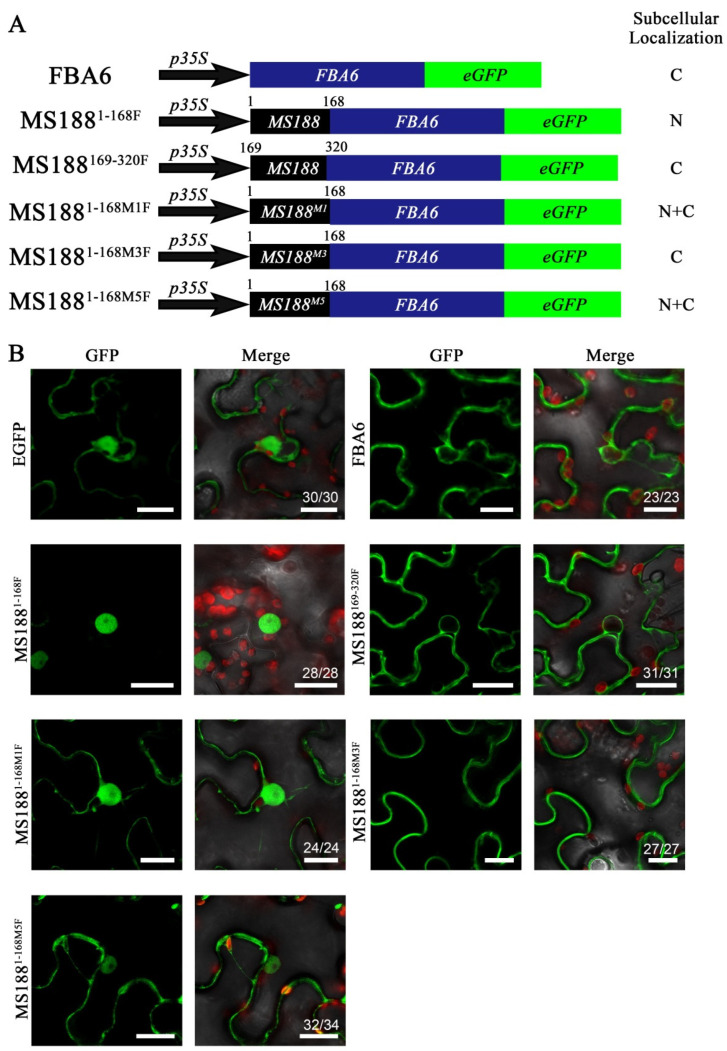
MS188^12–15^, MS188^18–22^, and MS188^96–107^ are the NLSs of MS188. (**A**) The cytoplasmic protein FBA6 was used to confirm the function of the MS188 NLSs: MS188^12–15^, MS188^18–22^, and MS188^96–107^. Linker peptides (PTPTPTPTP) were added between MS188 and FBA6. The chimeric proteins were fused with GFP and infiltrated into tobacco leaves. N, nucleus; C, cytoplasm. (**B**) The fluorescence of FBA6-GFP was detected only in the cytoplasm, but the MS188^1–168^ N-terminal fusion was transferred into the nucleus, whereas for the MS188^169–320^ fusion, the fluorescence was still distributed in the cytoplasm. Fusions with mutated MS188^1–168^ at the N-terminus, MS188^1–168M1F^-GFP and MS188^1–168M5F^-GFP, were distributed throughout the cytoplasm and the nucleus. MS188^1–168M3F^-GFP was detected only in the cytoplasm. Bars = 20 μm. The number in the Merge panel represents the statistical number of observations.

To investigate whether these putative NLSs indeed have nuclear localization, we used the fructose 1,6-bisphosphate aldolase FBA6, which has been reported to be a cytoplasmic protein [42], fused with GFP as a reporter (Figure 4A). The fluorescence signal of FBA6-GFP was completely located in the cytoplasm (Figure 4B). We further constructed truncated or mutated MS188 linked with the N-terminus of FBA6-GFP (Figure 4A). As the functions of MS188 and FBA6 are completely different, we added a linker peptide (PTPTPTPTP) to avoid interference between them [43]. MS188^1–168^-FBA6-GFP fluorescence was detected only in the nucleus, whereas MS188^169–320^-FBA6-GFP was located in the cytoplasm (Figure 4B). These results suggested that the NLS in MS188^1–168^ could enable FBA6 to enter the nucleus. The above results revealed that the putative NLSs of MS188 included AA12–15(M1), AA18–22(M3), and AA96–107(M5) (Figure 3). Among the point mutations, the MS188^M1^-FBA6-GFP and MS188^M5^-FBA6-GFP fluorescence signals appeared in the cytoplasm, but the signal was also still present in the nucleus (Figure 4B). This result indicated that mutation of these two sites could affect the NLS function of these vectors. However, the MS188^M3^-FBA6-GFP signal was completely localized in the cytoplasm rather than the nucleus, suggesting that AA18–22(M3) is essential for NLS functions to transport FBA6 into the nucleus (Figure 4B). Interestingly, the amino acid residues spanning AA18–22 contain three acidic amino acid residues instead of the basic amino acid residues in the traditional NLS.

### 3.5. The CDS Without NLS Sites Failed to Complement the Male Sterile Phenotype of ms188

**Figure 5:**
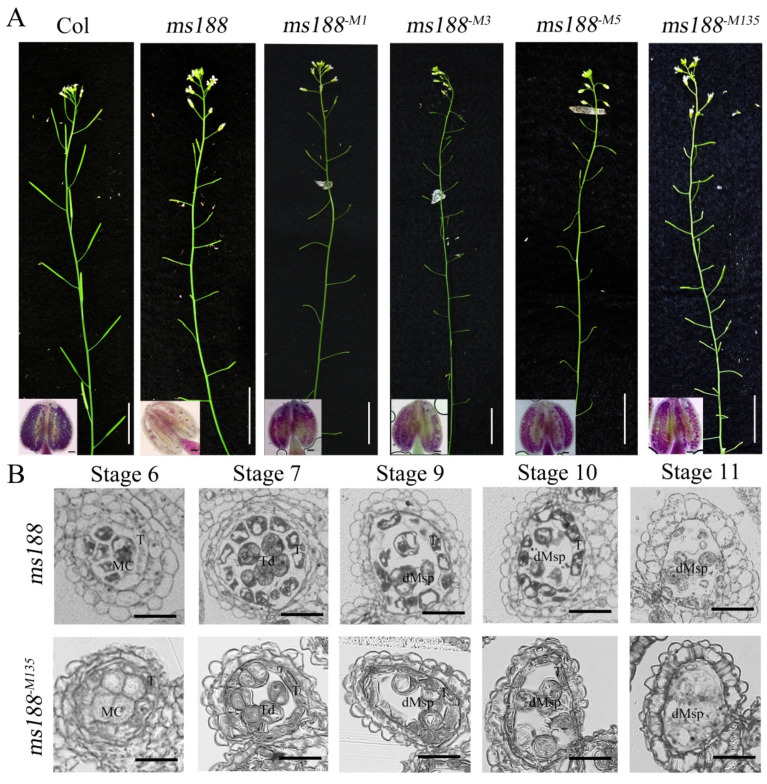
The function of MS188 was impaired when its NLS sites were mutated. (**A**) Phenotypes of the wild-type (Col), *ms188* mutant, and transgenic plants. A wild-type Col plant presented normal fertility. An *ms188* mutant presented very small siliques containing no seeds. All of the *MS188^-M1^*, *MS188^-M3^*, *MS188^-M5^*, and *MS188^-M135^* transgenic lines presented small siliques without seeds. Bars = 2 cm. Insets in A show the pollen grains in the anthers stained with Alexander’s stain. Bars = 50 μm. (**B**) Semithin sections of *ms188* and *MS188^-M135^* anthers at different stages. dMSp, degraded microspores; MC, meiocytes; T, tapetum; Td, tetrad.

The above tobacco transient expression assays revealed that the mutation of the NLS sites of MS188 led to diffuse subcellular localization into the cytoplasm (Figure 3). We further constructed the MS188 coding region with *MS188^-M1^*, *MS188^-M3^*, *MS188^-M5^*, and *MS188^-M135^*, with mutations at all three sites driven by the *MS188* native promoter and transformed them into *ms188*^+/−^ heterozygotes. We ultimately obtained five *MS188^-^^M1^* transgenic plants, seven *MS188^-M3^* lines, eight *MS188^-M5^* lines, and six *MS188^-M135^* lines in the *ms188*^−/−^ background. None of these transgenic plants recovered the male sterile phenotype of the *ms188* mutant (Figure 5A). Alexander staining revealed no pollen grains in the anther locules of *ms188* (Figure 5A); however, the transgenic plants still had many aborted pollen grains in the anther locules (Figure 5A). To further analyze anther development defects in the transgenic plants in detail, semithin sections of *MS188^-M135^* and *ms188* anthers were obtained. The *ms188* mutant exhibited obvious vacuolization of the tapetal layer at stage 7 (Figure 5B) [28]. The *MS188^-M135^* lines did not present such a strong defect phenotype in the tapetum layer (Figure 5B). However, their tetrads still failed to separate at stage 9, and the microspores gradually ruptured at stage 11 (Figure 5B). Therefore, we propose that disruption of the NLS of MS188 affects not only its subcellular localization but also the biological function of this transcription factor, resulting in a male sterile phenotype.

### 3.6. These NLSs Are Conserved in the R2R3-MYB Family in Arabidopsis

**Figure 6:**
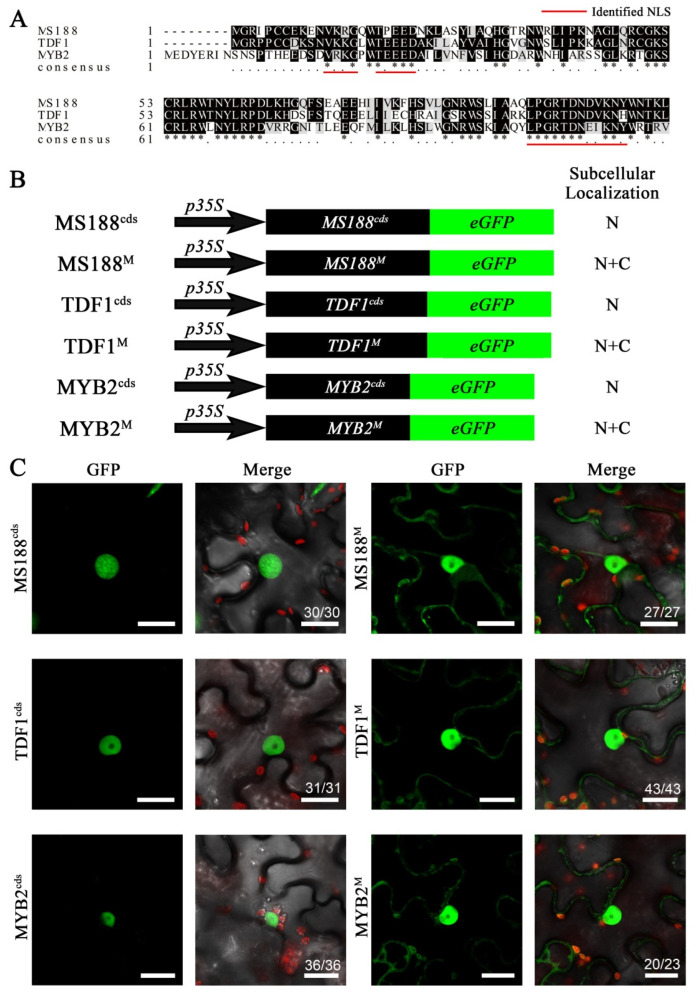
The NLS is conserved in R2R3-MYB family proteins. (**A**) Amino acid sequence alignment of the R2R3-MYB domain of three R2R3-MYB genes: MS188, TDF1, and MYB2. The refined NLS motif is marked by the red bar. Dark shading, identical residues; light shading, similar residues. * represents the identical animo acids between two sequences. (**B**) Point mutations of all the NLSs (MS188^12–15^, MS188^18–22^, and MS188^96–107^) in MS188, TDF1, and MYB2 were constructed and fused with GFP and then infiltrated into tobacco leaves. N, nucleus; C, cytoplasm. (**C**) The fluorescence of MS188-GFP, TDF1-GFP, and MYB2-GFP was detected only in the nucleus. Fluorescence of the point mutations MS188^M^-GFP, TDF1^M^-GFP, and MYB2^M^-GFP was distributed throughout the cytoplasm and the nucleus. Bars = 20 μm. The number in the Merge panel represents the statistical number of observations.

The R2R3-MYB domain is highly conserved across the whole Arabidopsis R2R3-MYB family [6]. Among the NLS sites we identified, AA12–15 and AA18–22 were located in the N-terminus of the R2 domain, and AA96–107 were located in the C-terminus of the R3 domain. These NLS motifs are highly conserved in these R2R3-MYB transcription factors (Figure 6A). Another R2R3-MYB transcription factor, TDF1, is essential for tapetal development and functions in anther genetic pathways [26]. MYB2 is involved in regulating the formation of axillary meristems (AMs) and plant aging, the functions of which differ widely from those of MS188 [44]. We chose these two R2R3-MYB proteins to examine the conservation of NLS function. Three mutation sites (MS188^12–15^, MS188^18–22^, and MS188^96–107^) were introduced into TDF1, MYB2, and MS188 (Figure 6B). The green fluorescence signals of wild-type MS188, TDF1, and MYB2 are completely localized to the nucleus, suggesting that these proteins function as transcription factors (Figure 6C). However, the GFP signals of MS188^M^, TDF1^M^ and MYB2^M^ were distributed in both the cytoplasm and the nucleus (Figure 6C), indicating that the nuclear entry function of these MYB proteins was affected. These results suggested that these NLS sites are highly conserved in the Arabidopsis R2R3-MYB family.

## 4. Discussion

### 4.1. The R2R3 Domain in MYB Family Functions in Binding DNA and Nuclear Localization

A transcription factor (TF) generally has four functional regions: DBDs, transcription regulation domains, oligomerization sites, and NLSs. As one type of TF, R2R3-MYB proteins can bind DNA via the R2R3-MYB domains [45]. Previous studies showed that MS188 could bind to the cis-element promoters of sporopollenin-related genes and activate their expressions [30,31]. However, the NLSs of MS188 have not yet been discovered. In this study, we found that the MS188^1–124^-GFP signal is located in the nucleus (Figure 2B), suggesting that this R2R3-MYB domain not only acts as a DNA-binding domain but also as an NLS site, which facilitates TF entry into the nucleus through the nuclear pore from the cell cytoplasm. A previous study revealed that AtMYB59 and AtMYB48 undergo alternative splicing of pre-mRNAs, producing different splice variants with varied expression patterns. Therefore, the authors suggested that both genes may have two basic amino acid regions that serve as NLSs in R3 repeats [46]. Here, our results with base mutations identified two NLS regions located at the N-terminus of R2-MYB and one at the C-terminus of R3-MYB in MS188 (Figure 3). We then chose the MS188^66–121^ fragment, including two predicted NLSs, and fused it into a modified DYT1-GFP vector. It has been reported that DYT1 is a non-nuclear TF during anther development [47]. We then observed the DYT1-GFP signals through the transient expression assay in tobacco leaves. However, the fluorescent DYT1-GFP did not express in either the nucleus or cytoplasm (Figure S1), suggesting that the NLS in the R3 domain may not solely participate in the process of nuclear localization. 

### 4.2. The NLS Region of MS188 Contains a New Amino Acid Sequence Conserved in the R2R3-MYB Family

The typical single-cluster type NLS is 126Pro-Lys-Lys-Lys-Arg-Lys-Val132, which is derived from the antigen of simian virus 40 (SV40) [34]. The predicted NLS is located at position 111KLKKL116, which is the MS188^M6^ position. However, the results of this study showed that the mutation of MS188^M6^ sites did not affect the nuclear localization of MS188 (Figure 3B), indicating that this sequence is irrelevant to nuclear localization. We then used segmented cloning and point mutation to determine three key NLS sites in MS188, which are located at amino acid residues 12–15, 18–22, and 96–107. These NLS sites are highly conserved in the Arabidopsis R2R3-MYB family (Figure 6A). The transient expression assays showed that mutation of these NLS sites would also affect the nuclear entry of TDF1 and MYB2 (Figure 6C). In addition, the sequence analysis showed that these NLS sites are highly conserved in MS188 homologous of various species, including moss, fern, and monocotyledonous and dicotyledonous plants (Figure S2), implying that these NLS sites may also play a crucial role in the nuclear localization of plant R2R3-MYB transcription factors. Previous studies showed that the typical NLS contains clusters with one or two alkaline amino acids (lysine and arginine) [34,35]. Among three NLS sites of MS188, several alkaline amino acids are present in AAs 12–15 and AAs 96–107: 13K, 14R, 100K, and 106K. Mutations of these amino acids obviously affected MS188 nuclear localization (Figure 3C), suggesting that these four alkaline amino acids are required for the nuclear entry of MYB family TFs. Interestingly, there is a highly conserved 20EED22 motif in AAs 18–22 (MS188^M2^ region), which consists of entirely acidic amino acids. Mutations in this region also disturbed the NLS function (Figure 3B), suggesting that this region is a new type of NLS site in R2R3-MYB transcription factors.

### 4.3. The Impact of the NLS Region of MS188 on Nuclear Entry and Function

Transcription factors are efficiently transported from the cytoplasmic synthesis site to the nucleus through the nuclear pore complex (NPC). Translocation through the NPC is mediated by importins, which are divided into subfamilies of import complex subunits IMPα and IMPβ [48]. The transcription factors act as the cargo proteins, which require the NLS amino acids to interact with importin α for nuclear entry [40,41]. In this study, we identified three NLS regions of MS188, an R2R3-MYB family member. These NLS sites could help the translocation of FBA6, a cytoplasmic protein, to the nucleus (Figure 4B), suggesting they are essential for protein nuclear entry. Our genetic evidence revealed that the MS188 protein without NLS sites failed to complement the male sterile phenotype of *ms188* (Figure 5), confirming that the normal function of MS188 depends on these NLS sites. A recent study showed that the NLS region of a class IV homeodomain leucine zipper (HD-Zip IV) transcription factor GL2 could interact with several IMPα isoforms [49]. In future, we will detect whether the translocation process of R2R3 MYB protein depends on the interaction between NLS and IMPα. In Arabidopsis secondary metabolism, MYB family members commonly interact with bHLH factors to regulate the downstream genes involved in plant development and growth [50,51,52]. During reproductive development, some bHLHs cannot enter the nucleus by themselves and depend on other TFs for nuclear entry, such as DYT1 [47]. MS188, a major activator, interacts with other bHLHs to function in the transcriptional regulatory pathway of downstream genes [30,31]. Therefore, we proposed that these NLSs of MS188 might play a general role in the nuclear localization of other bHLHs.

### 4.4. Conclusions

This research identified the NLS sites of the R2R3-MYB transcription factor in plants, which exhibit a bipartite cluster-type NLS located at the two ends of the R2R3-MYB domain, including a new type of NLS with acidic amino acids. These NLS sites are essential for the normal function of MS188. Subcellular localization in this study was performed utilizing a transient expression assay in the *Nicotiana benthamiana* epidermis. In the future, we will confirm these results in Arabidopsis native tissues.

## Data Availability

Data are contained within the article and Supplementary Materials.

## Supplementary Materials

The following supporting information can be downloaded at https://www.mdpi.com/article/10.3390/cells14070470/s1. Figure S1: The R3-MYB region of MS188 (AA 66–121) is not localized to the nucleus; Figure S2: Amino acid sequence alignment of the R2R3-MYB domain of MS188 homologous; Table S1: Primers used in the study.
