# Supplementary material for: Identification of Nuclear Localization Sequence (NLS) Sites in R2R3-MYB Transcription Factor Involved in Anther Development
Source: Cells. 2025 Mar 21;14(7):470. doi: 10.3390/cells14070470 (PMC11987959; doi:10.3390/cells14070470)
Supplement: Supplementary file 1 [file cells-14-00470-s001.zip › cells-3481557-supplementary.pdf]

Supplementary Materials for

Identification of nuclear localization sequence (NLS) sites in an R2R3-MYB transcription factor involved in anther development

This file includes:

Figure S1: The R3-MYB region of MS188 (66-121AA) is not located in the nucleus
Figure S2: Amino acid sequence alignment of the R2R3-MYB domain of MS188 homologous
Table S1: Primers used in the study

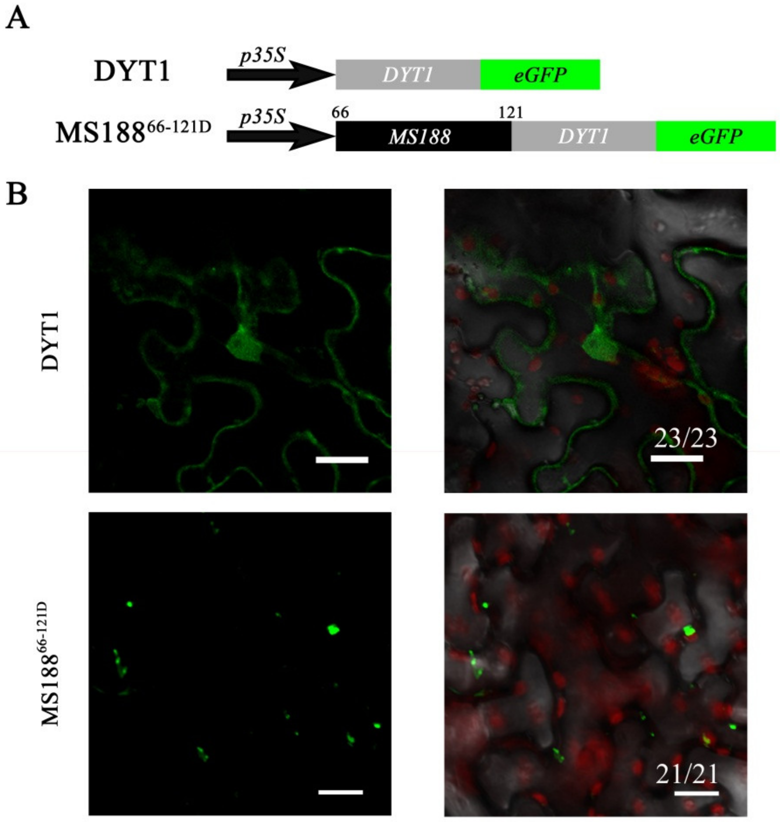

Figure S1. The R3-MYB region of MS188 (66-121AA) is not located in the nucleus
(A) MS188$^{66\text{-}121}$ was fused to the N-terminus of DYT1 and GFP and infiltrated into tobacco leaves. N, nucleus; C, cytoplasm. (B) Dotted fluorescence of MS188$^{66\text{-}121D}$-GFP was observed in the cytoplasm but was not detected in the nucleus. Bars = 20 µm. The number in the Merge panel represents the statistical number of observations.

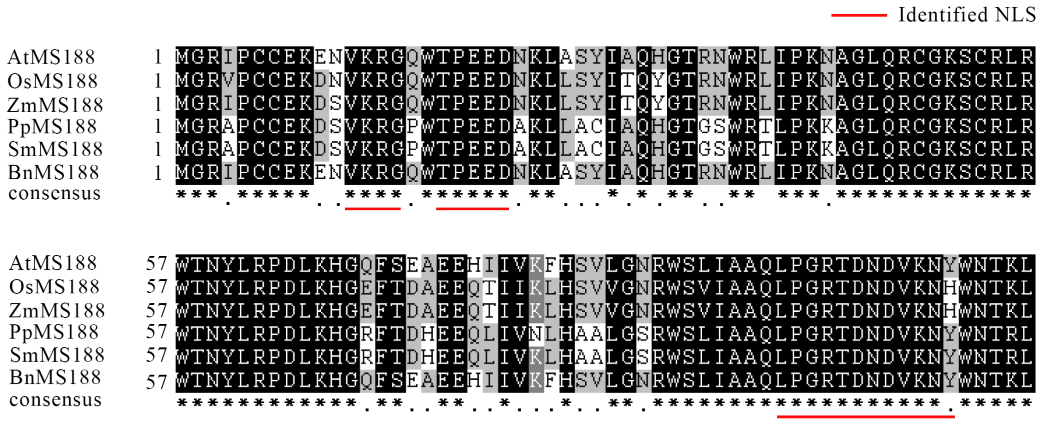

Figure S2. Amino acid sequence alignment of the R2R3-MYB domain of MS188 homologous.

Amino acid sequence alignment of the R2R3-MYB domain of AtMS188, OSMS188, ZmMS188, PpMS188, SmMS188, and BnMS188. The refined NLS motif is marked by the red bar. Dark shading, identical residues; light shading, similar residues. At, *Arabidopsis thaliana*; Os, *Oryza sativa*; Zm, *Zea mays*; Pp, *Physcomitrium patens*; Sm, *Selaginella moellendorffii*; Bn, *Brassica napus*.

Table S1 Primers used in the study

Primer ID	Sequence (5' to 3')
MS188-GFP-F	GCTCGGTACCCGGGGATCCATGGGTCGGATTCCATGTT
MS188-168-GFP-R	CCATGTCGACTCTAGAACGTTTCTTGGTGAGCAAG
MS188-169-F	GCTCGGTACCCGGGGATCCATGGTTGACCTAAACCAAATCAACTT
MS188-GFP-R	TCACCATGTCGACTCTAGAAACCATATGATTGATGAGATC
MS188-6ATG-GFP	GCTCGGTACCCGGGGATCCATGTGTTGTGAAAAGGAGAAT
MS188-11ATG-F	GCTCGGTACCCGGGGATCCATGAATGTGAAGAGAGGACAAT
MS188-16ATG-F	GCTCGGTACCCGGGGATCCATGCAATGGACTCCTGAAGAAG
MS188-21ATG-F	GCTCGGTACCCGGGGATCCATGGAAGACAACAAATTGGCTT
MS188-124-GFP-R	TCACCATGTCGACTCTAGACACCGGATCTATTCCCATTC
MS188-103-GFP-R	TCACCATGTCGACTCTAGAATCGTTGTCTGTCCGACCAG
MS188-12AAAA15-R	TGCTGCTGCTGCATTCTCCTTTTCACAACATGGA
MS188-12AAAA15-F	GCAGCAGCAGCACAATGGACTCCTGAAGAAGACA
MS188-18-F	ACTCCTGAAGAAGACAACAAATTG
MS188-W17A-R	GTCTTCTTCAGGAGTTGCTTGTCCTCTCTTCACATTCTC
MS188-18AAAAA22-GFP-R	TGCTGCTGCTGCTGCCCATTGTCCTCTCTTCACATTCT
MS188-18AAAAA22-GFP-F	GCAGCAGCAGCAGCAAACAAATTGGCTTCTTATATTGC
MS188-24AA25-GFP-R	ATAAGAAGCTGCTGCGTTGTCTTCTTCAGGAGTCCATT
MS188-24AA25-GFP-F	GAAGACAACGCAGCAGCTTCTTATATTGCTCAACATGGT
MS188-96A107-F	TTGATTGCGGCGCAAGCAGCAGCAGCAGCAGCAGCAGCAGCAGCAGCAGCATGGAACACGAAGCTG
MS188-96A107-R	CAGCTTCGTGTTCCATGCTGCTGCTGCTGCTGCTGCTGCTGCTGCTGCTTGCGCCGCAATCAA
MS188-95-NR	TTGCGCCGCAATCAAC
MS188-108-NF	TGGAACACGAAGCTGAAGAAG
MS188-109AAAAAAAA116-R	TGCTGCTGCTGCTGCTGCTGCTGCCCAATAATTTTTCACATCGTTGT
MS188-109AAAAAAAA116-F	GCAGCAGCAGCAGCAGCAGCAGCATCAGGAATGGGAATAGATCCG
35S-FBA6-G-F	GCTCGGTACCCGGGGATCCATGTCTTCCTTCACCTCCAAAT
35S-FBA6-G-R	TCACCATGTCGACTCTAGAGTACTTGTAATCCTTAACGTGAAGG
M168(L-FBA)-R	GAGGTGAAGGAAGACATAGGTGTAGGTGTAGGTGTAGGTGTAGGACGTTTCTTGGTGAGCAAGTG
M320(L-FBA)-R	GAGGTGAAGGAAGACATAGGTGTAGGTGTAGGTGTAGGTGTAGGAACCATATGATTGATGAGATC
MYB2-19R	ATCAGAATCTTCTTCATGTGTTGG
MYB2-31F	GCAATCCTAGTCAACTTCGTCTC
MYB2-103R	ATATTGCGCAATCTTCGACC
MYB2-116F	TGGAGAACTCGAGTCCAAAAG

MYB2-20-30-F	TGAAGAAGATTCTGATGCAGCAGCAGCACCATGGGCAGCAGCAGCAGCAGCAATCCTAGTCAACT
MYB2-20-30-R	AGTTGACTAGGATTGCTGCTGCTGCTGCTGCCCATGGTGCTGCTGCTGCATCAGAATCTTCTTCA
MYB2-104-115-F	GAAGATTGCGCAATATGCAGCAGCAGCAGCAGCAGCAGCAGCAGCAGCAGCATGGAGAACTCGAGTCC
MYB2-104-115-R	GGACTCGAGTTCTCCATGCTGCTGCTGCTGCTGCTGCTGCTGCTGCTGCTGCATATTGCGCAATCTTC
35S-MYB2-GFP-F	GCTCGGTACCCGGGGATCCATGGAAGATTACGAGCGAATAAA
35S-MYB2-GFP-R	TCACCATGTCGACTCTAGAATTATACGAATACGATGTCGTATCG
35S-TDF1-GFP-F	GCTCGGTACCCGGGGATCCATGGAAGACCTCCTTGTTG
35S-TDF1-GFP-R	TCACCATGTCGACTCTAGAATAATCGAAATCATTCAAGAGTTGA
TDF1-11R	ATTGGACTTGTCACAACAAGGA
TDF1-23F	GCTAAGATCCTTGCTTATGTTGC
TDF1-95R	CTTTCGTGCAATGGAAGACC
TDF1-108F	TGGAACACAAAGCTGAAGAAGA
TDF1-12-22-F	TTGTGACAAGTCCAATGCAGCAGCAGCACTCTGGGCAGCAGCAGCAGCAGCTAAGATCCTTGCTT
TDF1-12-22-R	AAGCAAGGATCTTAGCTGCTGCTGCTGCTGCCCAGAGTGCTGCTGCTGCATTGGACTTGTCACAA
TDF1-96-107-F	TTCCATTGCACGAAAGGCAGCAGCAGCAGCAGCAGCAGCAGCAGCAGCAGCATGGAACACAAAGCTG
TDF1-96-107-R	CAGCTTTGTGTTCCATGCTGCTGCTGCTGCTGCTGCTGCTGCTGCTGCTGCCTTTCGTGCAATGGAA
35S-DYT1-GFP-F	GCTCGGTACCCGGGGATCCATGGGTGGAGGAAGCAGATT
35S-DYT1-GFP-R	TCACCATGTCGACTCTAGATGGATTGCTTCTCATAACTTCC
35S-M66-DYT1-F	GACTGAGCTCGGTACCATGAAACATGGCCAGTTCTCGG
35S-M121-DYT1-R	TGCTTCCTCCACCCATTATTCCCATTCCTGACAACTTCT
ProMS188-F	AGCTATGACCATGATTACGTAATATCTATCCAAAGAAAATC
ProMS188-R	CCCCGGGTACCGAGCTCTTCTTCTTTCTTTCTTTCTAGTTTT
pM-MS188-F	AAAACTAGAAAGAAAGAAAGAAGAAATGGGTCGGATTCCATGTT
pM-MS188-R	TAGAGGATCCCCGGGTACCGAGCTCTCAAACCATATGATTGATGAGATC
MS188-1314F	ATGTGGCAGCAGGACAATGGACTCCTGAAGAAGA
MS188-1314R	TTGTCCTGCTGCCACATTCTCCTTTTCACAACATG
MS188-106F	ACAGACAACGATGTGGCAAATTATTGGAACACGAAGCT
MS188-100R	TGCCACATCGTTGTCTGTTGCACCAGGAAGTTGCGCCG
MS188-1215-F	GCAGCAGCAGCACAATGGACTCCTGAAGAAGACA
MS188-1215-R	TGCTGCTGCTGCATTCTCCTTTTCACAACATGGA
